# Ovarian Leydig Cell Hyperplasia: An Unusual Case of Virilization in a Postmenopausal Woman

**DOI:** 10.1155/2014/762745

**Published:** 2014-06-19

**Authors:** Jaya M. Mehta, Jeffrey L. Miller, Anthony J. Cannon, Stacey K. Mardekian, Lawrence C. Kenyon, Serge A. Jabbour

**Affiliations:** ^1^Division of Endocrinology, Diabetes & Metabolism, Jefferson Medical College of Thomas Jefferson University, 211 South 9th Street, Suite 600, Philadelphia, PA 19107, USA; ^2^Private Practice, 3836 Quakerbridge Road, Suite 206, Trenton, NJ 08619, USA; ^3^Department of Pathology, Jefferson Medical College of Thomas Jefferson University Hospital, 285 Main Building, 132 South 10th Street, Philadelphia, PA 19107, USA; ^4^Department of Pathology, Jefferson Medical College of Thomas Jefferson University Hospital, 280 Main Building, 132 South 10th Street, Philadelphia, PA 19107, USA

## Abstract

*Objective*. To report an unusual case of ovarian Leydig cell hyperplasia resulting in virilization in a postmenopausal woman. *Methods*. Patient's medical history and pertinent literature were reviewed. *Results*. A 64-year-old woman presented with virilization with worsening hirsutism, deepening of her voice, male musculature, and male pattern alopecia. Her pertinent past medical history included type 1 diabetes, hyperlipidemia, and hypertension. Her pertinent past surgical history included hysterectomy due to fibroids. On further work-up, her serum total testosterone was 506 ng/dL (nl range: 2–45) and free testosterone was 40 pg/mL (nl range: 0.1–6.4). After ruling out adrenal causes, the patient underwent an empiric bilateral oophorectomy that showed Leydig cell hyperplasia on pathology. Six weeks postoperatively, serum testosterone was undetectable with significant clinical improvement. *Conclusion*. Postmenopausal hyperandrogenism can be the result of numerous etiologies ranging from normal physiologic changes to ovarian or rarely adrenal tumors. Our patient was found to have Leydig cell hyperplasia of her ovaries, a rarely reported cause of virilization.

## 1. Introduction

In a postmenopausal woman, evaluation of virilization includes ruling out a variety of causes before ovarian sampling or empiric oophorectomy. Mild hirsutism and alopecia in postmenopausal women can be a normal physiological response: with a reduction of ovarian follicles, there is a diminished estrogen and progesterone secretion, thus increasing the impact of androgens on sebaceous glands and hair follicles resulting in transformation of vellus to terminal hair follicles [1]. Apart from the normal physiological changes leading to hirsutism, there are various conditions that need to be ruled out and/or treated that result in virilization in postmenopausal patients. Included in these are polycystic ovary syndrome (PCOS), congenital adrenal hyperplasia (CAH), Cushing's syndrome, benign and malignant androgen-secreting ovarian and adrenal tumors, and iatrogenic causes [1]. This case report is of a patient with a rare but relevant cause of postmenopausal hyperandrogenism: ovarian Leydig cell hyperplasia. This pathology, previously reported in at least seven cases, results in excess testosterone secretion and the virilizing symptoms of the patient, a relationship elucidated by disappearance of symptoms after bilateral salpingooopherectomy.

## 2. Case Presentation

A 64-year-old woman presented to the endocrinology office as a referral for evaluation of increasing hirsutism. She had a history of irregular menses and facial hirsutism since menarche, but since menopause at age 50, she noticed worsening hirsutism, diffuse hair loss, deepening of her voice, increased muscle mass, and increased libido.

Her past medical history included type 1 diabetes, hyperlipidemia, and hypertension. The patient's past surgical history was significant for a total abdominal hysterectomy for fibroids. Her obstetrical history was significant for two live births, both products of ovulation induction. Her medications at the time of the visit included insulin glargine, insulin aspart, nifedipine, atorvastatin, and valsartan. Her family history was significant for a mother with heart disease and hypertension and a father with stroke and hypertension. Her social history was significant for two drinks a day and smoking a half-a-pack per day for thirty years.

On physical exam, her blood pressure was 112/74 mm Hg and a body mass index of 24.9 kg/m^2^. She had male pattern alopecia with mild male musculature and facial hirsutism on her chin and upper lip. She was neither cushingoid nor acromegalic. Her external female genitalia were grossly normal in appearance and her bimanual and rectovaginal exam failed to demonstrate any masses.

Pertinent laboratory work-up at a previous office showed the following: serum total testosterone: 588 ng/dL (nl range: 2–45), 17-hydroxyprogesterone: 129 ng/dL (nl range: <45), cortisol: 14 mcg/dL (nl range: 4–28.6), ACTH: 5 pg/mL (nl range: 6–50), LH: 43 mIU/mL (nl: 7.7–58.5), and FSH: 41 mIU/mL (nl range: 25.8–135). A basic metabolic profile was within normal limits. Her HbA1c was 6.2%. Pelvic and transvaginal ultrasound showed no ovarian masses. On transvaginal ultrasound, her left ovary measured 2.8 × 2.3 × 2.9 cm and her right measured 2.3 × 1.7 × 1.6 cm, and no adnexal masses or free fluid collections were visualized. Magnetic resonance imaging (MRI) of her abdomen and pelvis showed normal ovaries with few tiny cysts of no significance and her adrenals were both slightly enlarged without any masses. MRI of her pituitary was normal.

Repeat laboratory work at our office showed the following: serum cortisol: 8.6 mcg/dL at baseline and 39.2 mcg/dL after cosyntropin stimulation, ACTH: 12 pg/mL (nl range: 6–50), LH: 32 mIU/mL (nl range: 7.7–58.5), FSH: 49 (nl range: 25.8–135), DHEAS: 68 mcg/dL (nl range: 45–430), estradiol: 56 pg/mL (nl range: <5.0–54.7), aldosterone: 8 ng/dL (nl range: 4–31), total testosterone: 506 ng/dL (nl range: 2–45), free testosterone: 40 pg/mL (nl range: 0.1–6.4), 17-hydroxyprogesterone at baseline: 97 ng/dL (nl < 45) going up to 539 ng/dL at 60 minutes after cosyntropin, 11-deoxycortisol: 30 ng/dL (nl < 32) at baseline going up to 270 ng/dL after cosyntropin, androstenedione: 2.37 ng/mL (nl: 0.130–0.820), alpha-fetoprotein: 10.3 ng/mL (nl range: 0.0–7.7), and inhibin: 1.1 pg/mL (nl < 7.9). Her karyotype analysis came back as 46, XX.

In order to rule out late-onset CAH, we performed a dexamethasone suppression test where the patient was on dexamethasone 0.5 mg every 6 hours for 5 days and then underwent repeat serum testosterone, 11-deoxycortisol, and 17-hydroxyprogesterone. After dexamethasone, the patient's total testosterone was 646 ng/dL (nl range: 2–45) and her free testosterone was 60.1 pg/mL (nl range: 0.1–6.4). With nonsuppressible total testosterone, late-onset CAH could be ruled out as a cause of her hyperandrogenism in conjunction with absence of adrenal tumor on imaging. Therefore, we proceeded with empiric bilateral salpingooopherectomy as she deferred selective sampling. However, the high 11-deoxycortisol postcosyntropin could indicate a mild coexistent late-onset CAH due to 11-hydroxylase deficiency.

**Figure 1:**
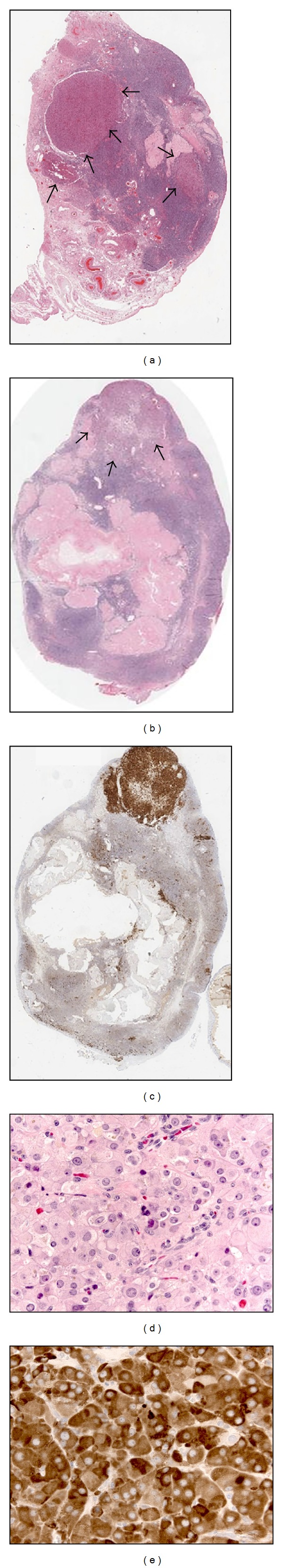
Whole-slide sections of left (a) and right (b) ovaries display multiple eosinophilic nodules of Leydig cells (arrows). Immunohistochemistry for inhibin A highlights the nodules and identifies additional small clusters of Leydig cells (c, whole-slide section, right ovary). The nodules are composed of Leydig cells, which are polygonal with abundant granular eosinophilic cytoplasm and large vesicular nuclei (d, H&E, 400x). The Leydig cells display strong cytoplasmic staining with inhibin A (e, 400x).

The surgical pathology revealed that both the right and left ovaries contained multiple nodules and small clusters of bland showing Leydig cells along with unremarkable fallopian tubes. There was no significant mitotic activity and no evidence of a concomitant neoplastic stromal component, only normal appearing ovarian stroma. None of the nodules was large enough to distort the size and shape of the ovaries nor was there evidence of a large dominant nodule from which one might suspect the other nodules could have arisen. Immunochemistry for inhibin A was strongly positive in the Leydig cell nodules and helped to confirm the presence of smaller clusters of Leydig cells interspersed within the ovarian stroma (Figure 1). The presence of multiple bilateral nodules of Leydig cells is most compatible with Leydig cell hyperplasia rather than neoplasia. Six weeks postoperatively, serum testosterone was undetectable with significant clinical improvement.

## 3. Discussion

The patients' history of long-standing irregular menses and worsening hirsutism after menopause along with subsequent deepening voice, male musculature, and male alopecia, all accompanied by very high serum testosterone levels, prompted us to rule out late onset (CAH) and/or a small ovarian arrhenoblastoma. Before undergoing an oophorectomy, we wanted to make sure her high testosterone was not solely due to CAH.

Her long-standing history of hirsutism, irregular menses, recent lab findings other than her very high testosterone, and the slightly enlarged adrenals on imaging could be explained by late-onset CAH. An adrenal mass was ruled out as the etiology because adrenal masses secreting testosterone are big (above 1 cm) and usually cannot be missed by MRI. High testosterone levels (>150–200 ng/dL) such as hers can be seen in simple virilizing or salt losing forms of CAH; however, these forms of CAH are associated with adrenal insufficiency and high ACTH, both of which the patient did not have.

Small ovarian arrhenoblastomas are known to be small enough that imaging will not reveal these tumors leading to the need for ovarian sampling or bilateral oophorectomy. Since our patient had already undergone menopause, we recommended an oophorectomy rather than ovarian sampling. Ovarian sampling is very difficult and invasive and even at the best centers, sampling can accurately localize the ovarian tumor only 66% of the time [2].

Histologic examination of the patient's ovaries revealed multiple small bilateral nodules and interspersed small clusters of Leydig cells that were immunohistochemically positive for inhibin A. In this clinical context, Leydig cell hyperplasia is most likely given the small size of the nodules, a lack of a dominant nodule, and no evidence of metastatic disease. The fact that the patient's serum testosterone returned to normal following bilateral salpingooopherectomy also supports this contention since any metastatic tumor deposits would be expected to maintain an elevated serum testosterone. While inhibin expression is characteristic of Leydig cells, it is not specific. Inhibin A expression has been observed in many normal tissues and tumors; however, in the presence of the appropriate morphologic changes and clinical context, it can be an extremely useful adjunct for proper pathologic diagnosis [3]. Ultimately, ovarian Leydig cell hyperplasia was the etiology of our patient's symptoms. Though this pathology can affect both pre- and postmenopausal women, in our review of the literature, the cases reported have been of postmenopausal women. The cases tend to have similar presentations to our patient with a similar work-up and, ultimately, a bilateral salpingooopherectomy as the final treatment. Taylor et al. reported the first case of ovarian Leydig cell hyperplasia in 2000. The hyperplasia was characterized by small clusters or individual Leydig cells interspersed within the ovarian cortex in contrast with the more nodular growth pattern in our patient. Sequencing of the patient's LH receptor gene showed no abnormalities helping to clarify why the virilizing symptoms subsided after bilateral salpingooopherectomy [4]. Through a case of Leydig cell hyperplasia, Hofland et al. found that though DHEAS and imaging have high specificity in detecting adrenal causes of hyperandrogenism in postmenopausal patients, these tests are unable to detect ovarian disease [5]. Ali et al. presented a similar case of a postmenopausal woman with virilization. High testosterone was found in both ovarian and adrenal veins resulting in a unilateral adrenalectomy without resolution of symptoms; then, the patient received a bilateral oophorectomy leading to significant clinical and laboratory improvement [6]. Zafrakas et al. reported a case of premenopausal Leydig cell hyperplasia presenting as unilateral, grossly visible, circumscribed mass versus cases of Leydig cell hyperplasia in postmenopausal women presenting with bilateral, diffuse microscopic aggregates. They noted that this pathology in postmenopausal women was benign, making a bilateral salpingooopherectomy an appropriate cure [7]. Of note, Yoon et al. reported a case of Leydig cell hyperplasia in a 65-year-old patient whose only symptom was lumbago. Unlike our case, patient's Leydig cell hyperplasia was found within a simple cellular fibroma of her left ovary. The patient did not have androgenic effects of Leydig cell hyperplasia making it a nonfunctional proliferation [8]. This shows a difference in distribution of the Leydig cell hyperplasia and, thus, a difference in phenotype. Tutzer et al. published a similar case to ours in which a 79-year-old woman with hirsutism underwent a full work-up to rule out an adrenal tumor. Ultimately, surgery cured the patient's virilizing symptoms and ovarian Leydig cell hyperplasia was found [9]. With more of these rare cases of bilateral Leydig cell hyperplasia being reported in a very specific patient population, a new treatment option arises for those who have ruled out adrenal and/or ovarian causes.

## 4. Conclusion

Though at least seven cases have been described of this pathology, it is important to take note of our patient's presentation and final diagnosis for future work-ups in similarly presenting patients. With exclusion of adrenal origin and other obvious ovarian disease, virilization in postmenopausal women may be the result of Leydig cell hyperplasia for which a bilateral salpingooopherectomy or bilateral oophorectomy is the cure. A hyperandrogenic phenotype has a detrimental effect on the quality of life in postmenopausal patients as increased facial hair and loss of scalp hair may cause emotional and psychological distress [1]. It is important to work these patients up appropriately and understand the etiologies leading to hyperandrogenism to help alleviate the stresses associated with menopause and aging.
